# Open vs. Percutaneous Achilles Tendon Repair: Experience of Single Orthopedic Institute with Long-Term Follow-Up

**DOI:** 10.3390/medicina60091382

**Published:** 2024-08-23

**Authors:** Gaetano Caruso, Edoardo Gambuti, Achille Saracco, Elisa Spadoni, Elena Corso, Ilaria Pinotti, Alessandro Pisano, Leo Massari

**Affiliations:** 1 Department of Neurosciences and Rehabilitation, University of Ferrara, c/o “S. Anna”, via Aldo Moro 8, 44124 Ferrara, Italy; crsgtn@unife.it (G.C.); gambutiedoardo@gmail.com (E.G.); elisaspadoni7@gmail.com (E.S.); elenacorso14@gmail.com (E.C.); ilariapinotti1@gmail.com (I.P.); pisanomd@gmail.com (A.P.); 2 Department of Translational Medicine and for Romagna, University of Ferrara, c/o “S. Anna”, via Aldo Moro 8, 44124 Ferrara, Italy; msl@unife.it

**Keywords:** Achilles tendon, tendon ruptures, Tenolig^®^ repair system, tendon open repair, traumatology, trauma surgery, sport lesion

## Abstract

*Background and Objectives*: There are numerous techniques for the surgical treatment of Achilles tendon lesions described in the literature, and it is possible to distinguish repair techniques as either open surgery or percutaneous repair techniques. Both approaches have advantages and disadvantages. With this retrospective study, we aim to analyze the incidence of re-ruptures and other complications, return to sport and overall quality of life at a long-term follow-up in the treatment of acute ATRs, comparing the results of percutaneous repair with those of open repair. *Materials and Methods*: This is a retrospective study on a consecutive series of patients with complete tear of the AT who were managed through a surgical approach by the Operative Unit of Orthopaedics and Traumatology of Sant’Anna University Hospital (Ferrara, Emilia-Romagna, Italy) between April 2014 and December 2021. Patients were treated with a percutaneous or an open technique according to the surgeon’s preference without randomization. *Results*: We considered 155 patients who met the established inclusion criteria. Of these, 103 (66.45%) patients underwent percutaneous treatment with the Tenolig^®^ system, and 52 (33.55%) underwent open surgery, with an average ATRS in the first group of 92.5 compared to an average ATRS value of 82 in patients treated with the open technique. *Conclusions*: In our experience, following overlapping rehabilitation protocols in all patients included, we observed that the Tenolig^®^ repair system led to a better ATRS at long-term follow-up, with comparable complication rates to open surgery.

## 1. Introduction

Despite the increasing incidence of Achilles tendon ruptures (ATRs) and the growing interest of the medical community towards them, today, we still lack a widely accepted consensus about the optimal way to treat and manage such injuries [1]. Regarding acute ATRs, it is still under debate if these should be treated conservatively or surgically, with both options presenting advantages and disadvantages. Although surgical management exposes patients to the typical peri-operative risks (such as wound infections, deep infections, sural neuropathy, etc.), a lower re-rupture rate is also reported in comparison to the conservative strategy [1]. Many surgical approaches have been described over the years for Achilles tendon (AT) repair, but two main groups of procedures and techniques can be identified: open AT repair and percutaneous surgery. Open repair allows the surgeon to fully visualize the lesion and the tendon stumps, to have higher control of the other tissues such as the paratenon and, if needed, to perform additional procedures such as augmented repairs (e.g., using the Plantaris Muscle tendon), tendon transfers, etc. [2]. On the other hand, the open approach can expose patients to an increased risk of wound dehiscence, as well as superficial and deep infection [3]. Percutaneous surgery offers a minimally invasive way to repair ruptured Achilles tendons, yet a higher risk of sural neuropathy is reported [3,4]. The surgical indication regarding which technique to use is mainly based on the site of the lesion, the type of lesion and the patient’s characteristics, but also, significantly, on the surgeon’s preferences. For this reason, there is currently no real consensus in the literature towards either of the two techniques mentioned.

With the present work, we aim to analyze the incidence of re-ruptures, other complications, return to sport and overall quality of life at long-term follow-up in the treatment of acute ATRs, comparing the results of percutaneous repair with those of open repair.

## 2. Materials and Methods

This is a retrospective study on a consecutive series of patients with complete tear of the Achilles tendon (AT) who were managed through a surgical approach by the Operative Unit of Orthopaedics and Traumatology of Sant’Anna University Hospital (Ferrara, Emilia-Romagna, Italy) between April 2014 and December 2021. Patients were treated with a percutaneous or on open technique according to the surgeon’s preference without randomization. All surgeries were performed by two sports trauma surgeons with more than 15 years of experience.

The inclusion criteria were acute Achilles tendon rupture (time since rupture of less than 21 days), an age of over 18 years, complete data and the use of an open suture technique or percutaneous repair. We established a minimum clinical follow up of 48 months to be able to include a patient in the study. The exclusion criteria were chronic tendon rupture (time since rupture longer than 21 days), recurrent tendon rupture, proximal rupture at the muscle–tendon junction, distal tendon detachment and incomplete data.

The percutaneous treatment option was excluded in lesions that occurred more than 8 days ago and in lesions involving some portions of the tendon structure, in particular, lesions present less than 3 cm from the proximal myotendinous junction and lesions present less than 4 cm from the calcaneal insertional portion. In these situations, it was decided that open treatment would be performed.

The data taken into account were: gender, age, laterality, situational pattern, Achilles tendon total rupture score (ATRS), the sporting activity practiced, return to the same sport (same level, higher level or lower level), return to a different sport (explosive or non-explosive), complications with an effect on function (re-breaking, deep infections), complications without an effect on function (superficial infection, sural neuropathy, deep vein thrombosis (DVT), algodystrophy, clinical symptoms, follow up and operative time.

For the clinical evaluation of patients, we used a score called the Achilles Tendon Total Rupture Score (ATRS). The ATRS is the only patient-reported outcome measure (PROM) developed for the specific outcome assessment of an Achilles tendon rupture [5]. The ATRS consists of 10 items investigating the patient’s limitations and difficulties; in particular, it investigates in depth the outcomes of trauma by considering parameters such as muscle strength, stiffness, the perceived degree of fatigue and pain, analyzing these factors in various daily activities.

The Italian version of the ATRS demonstrated equivalent assessment capabilities compared to the original version. This makes this score valid for assessing the functionality of patients following ATRs [6].

Each item is given a score according to an 11-point Likert scale (0–10). A score of 0 corresponds to severe limitation and 10 to no limitation. The result, given by the sum of the items, varies from a score of 0 to 100, where 0 indicates serious symptoms and severe limitations, while 100 indicates an absence of symptoms and full functionality.

### 2.1. Open and Percutaneous Surgical Techniques

In patients treated with the percutaneous suture technique, the Tenolig^®^ suture system (Intrauma S.p.a, Rivoli, TO, Italy) was used for all patients. Also, in this case, the patient was positioned prone on an operating bed with a pad under their ankle, but in no patients was a tourniquet used. This Tenolig^®^ technique consists of passing two sutures with preloaded needles into which small harpoons are embedded. The entry points are about 6 cm proximal to the tear on the posterolateral side of the tendon. The exit points are on the posterolateral side of the tendon over the retromalleolar space, 4 to 5 cm below the tear. The surgeon passes the two needles percutaneously in a proximal to distal direction, through the ruptured tendon. The harpoons attach themselves into the upper portion of the tendon. The sutures are pulled downwards to bring the two ruptured tendon ends together, with the foot in an equinus position, and are then locked with polyethylene disks against the skin.

In all patients who underwent an open suture technique, a Krackow suture repair technique was employed using a no.2 Fiber wire. The patient was positioned prone on the operating bed with a pad under their ankle. In some cases, depending on the surgeon’s preference, a tourniquet was used. One Fiber wire was passed through the proximal portion of the tendon that required repairing. Five running/locking loops were passed through the tendon proximal to the site of the injury. The suture was then passed to the opposite side of the tendon, and a further 5 loops of running/locking were performed on the repair site. The operation was repeated in the distal portion of the tendon. During the tension of the sutures, and in particular, in the final phase, the foot was kept in an equinus position to facilitate bringing the edges of the tendon closer together. If possible, an accurate suture of the paratenon was also performed with detached Vicryl 3-0 stitches to restore this functional unit. Finally, a suture of the subcutaneous tissue was performed with 2-0 Vicryl suture thread and a suture of the skin using a mechanical stapler with metal clips.

### 2.2. Postoperative Management

Patients in both groups received a standard treatment with immobilization in a below-knee cast for 3 weeks and were non-weightbearing with crutches. After removal of the cast, partial weightbearing with crutches was allowed. After 3 weeks, patients were encouraged to perform unloaded plantar flexion exercises several times per day. Forty-five days after the surgery, the Tenolig was removed. Full weightbearing with normal shoes was allowed 45 days after the surgery in both groups. A gradual resumption of sporting activity was permitted 90 days after surgery.

All patients, except for those being treated with anticoagulants, underwent prophylactic therapy with 1 vial of Enoxaparin 4000 IU/day until they resumed physiological walking.

### 2.3. Statistical Analysis

Categorical data are expressed as absolute numbers and percentages. The statistical analysis of categorical variables was performed using Pearson’s χ^2^ test or Fisher’s exact test. The statistical analysis of continuous variables was performed using Student’s *t*-test for normally distributed variables and a Mann–Whitney U-test in the case of non-normal distribution. MedCalc^®^ Statistical Software version 19.8 (MedCalc Software Ltd., Ostend, Belgium) was used for statistical analyses, and the significance level was set to *p* < 0.05.

## 3. Results

**Figure 1:**
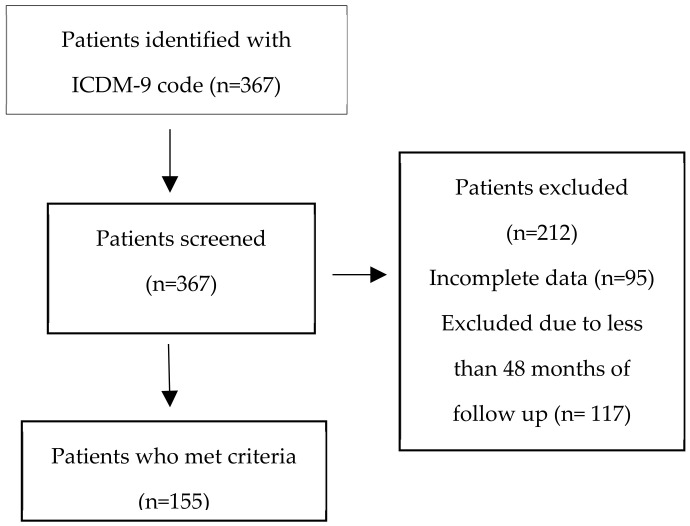
This figure represents the selection criteria and the final study population.

Of the 367 ATRs, only 155 met the inclusion criteria (Figure 1). Of these, 143 (92.3%) were male and 12 (7.7%) female. A total of 75 (48.4%) patients had a left tendon injury, while 80 (51.6%) had a right tendon injury. A total of 103 (66.45%) patients underwent percutaneous treatment with the Tenolig^®^ system, and 52 (33.55%) underwent open surgery. The mean age was 48.30 ± 14.75 years. A total of 81 (52%) cases were directly caused by sports injuries. In contrast, 74 injuries (48%) originated from accidental events, such as stair falls or traffic accidents. The mean ATRS was 91.2 ± 12.5. The mean follow-up was 72.3 ± 31.6 (Table 1).

### 3.1. Achilles Tendon Total Rupture Score (ATRS)

Patients treated with the percutaneous technique had a mean ATRS of 92.5 ± 10.0 versus a mean score of 82 ± 7.5 for patients treated with the open one (*p* = 0.156).

### 3.2. Procedure Duration

Patients undergoing the percutaneous technique had a mean operation duration of 19.4 ± 7.6 min. In contrast, patients undergoing the open technique had a surgery duration of 71.02 ± 26.70 min (*p* < 0.001). This result is statistically significant.

### 3.3. Complications

Analyzing the complications observed in our population, we report 12 complications for patients undergoing percutaneous surgery and 9 complications for those treated with open surgery.

In patients treated with the percutaneous technique with Tenolig^®^, we observed seven (6.8%) re-ruptures. In patients treated with the open technique, there were two (3.8%) re-ruptures. The *p*-value in the comparison between the two groups is *p* = 0.507, and this result is therefore statistically not significant.

In the group treated with the percutaneous technique, we observed two superficial infections, two cases of temporary paralysis of the sural nerve and one case of deep vein thrombosis. On the other hand, in the group treated with the open technique, we found four superficial infections, two cases of temporary paralysis of the sural nerve and two deep infections. By performing a statistical comparison between the two groups we observed the following:

♢ *p*-value = 0.991 for temporary paralysis of the sural nerve;
♢ *p*-value = 0.183 for superficial infections;
♢ *p*-value = 0.104 for deep infections.

The statistical analysis of complications reported only non-statistically significant values (Table 2).

### 3.4. Return to Sport

**Figure 2:**
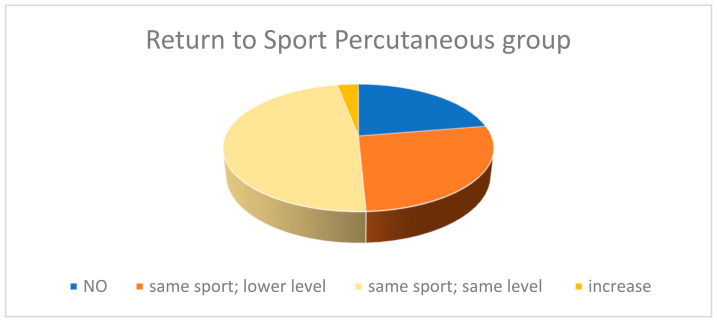
This figure represents the qualitative level of return to sporting activity in the group treated with the percutaneous technique.

In the percutaneous group, 67 (65%) patients practiced sport before sustaining ATRs. Of these, 18 (26.7%) returned to the same sport but at a lower level than pre-injury. A total of 32 (47.8%) continued to practice sport at the same level as before, while 2 (3.0%) increased their level of sporting activity compared with the pre-injury period. Only five patients (7.5%) performed an explosive sport. All five were not able to resume an explosive type of sport. Finally, 15 (22.4%) no longer played any type of sport post-injury (Figure 2).

**Figure 3:**
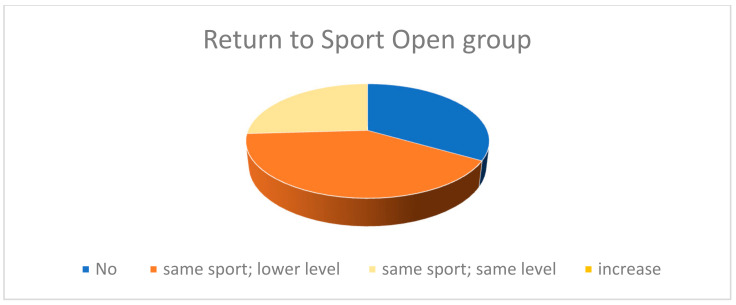
This figure represents the qualitative level of return to sporting activity in the group treated with the open technique.

In the open surgery group, 27 (51.9%) patients practiced sport before sustaining ATRs. Of these, 11 (40.7%) reported playing the same sport but at a lower level than pre-injury, and 7 (25.9%) maintained the same level as in the pre-injury period. None increased their level from the pre-injury period. Two (7.4%) patients changed from explosive to non-explosive sports. Nine (33.3%) patients decided to stop playing sports after the injury (Figure 3). 

No statistically significant differences were found between the two groups.

## 4. Discussion

There are uncertainties about the best treatment for acute Achilles tendon ruptures. The recent systematic review conducted by Amendola F. et al. [7] regarding treatment options identified the surgical approach as the most suitable solution, allocating conservative treatment only to patients with comorbidities or minimal functional requirements. Furthermore, comparable results seem to emerge from this work, with minimally invasive repair and accelerated functional rehabilitation.

Many surgical approaches have been described over the years for AT repair. Two main groups of procedures and techniques can be identified: open AT repair and percutaneous AT repair [2]. However, no technique has been proven to be the best [8]. ATRs have a high complication rate. In fact, more than 1 in 10 patients develop a postoperative complication [9]. Open AT repair is associated with a higher risk of superficial and deep infections, and a higher risk of ankle stiffness [10], while percutaneous surgery is associated with a greater risk of temporary sural nerve palsy [11]. Earlier studies did not find an increased re-rupture rate with the Tenolig^®^ system [12]. However, a higher re-rupture rate was found in more recent studies compared to the open technique. Laboute E et al. found a 22% re-rupture rate following ATR repair with the Tenolig^®^ system. On the other hand, the re-rupture rate of the open technique was only 1.3% [13]. Other studies confirm these data [14,15]. In our study, the re-rupture rate was higher in the percutaneous group compared to the open one, although not statistically significant. However, the re-rupture rate was significantly lower than that found in the literature [14,15]. These data, compared to the more recent literature, may be related to the relatively advanced age of our patients (48.30 ± 14.75). In fact, at 2–3 months, patients face the highest risk of re-rupture after Tenolig^®^ repair. This period coincides with the gradual resumption of physical activity. Older patients have lower functional demand, thus reducing the risk of re-ruptures in this period. Any comorbidities present in older subjects did not limit the physiotherapy process or require changes to the protocols applied.

Moreover, the Tenolig^®^ repair system is not as simple as it seems. In fact, Soubeyrand M et al. [16] showed that only in 45% of cases was the Tenolig^®^ correctly positioned. To improve Tenolig^®^ positioning some authors have suggested the use of intra-operative ultrasound [17]. In our clinical practice, only one case was treated with this aid but, limited to the single procedure, our feedback agrees with what was stated by Yongliang Y et al. [18] following their experience.

All these data demonstrate the long learning curve of this technique. In this study, both surgeons had more than 15 years’ experience with this technique. This could also explain the reduced incidence of re-rupture.

Percutaneous repair is a closed procedure originally described by Ma and Griffith [19]. Since that time, several studies have compared clinical outcomes between open and percutaneous surgery. Currently, the percutaneous approach involves various techniques and, as in our case, the use of devices designed to obtain a more functional reconstruction. In 2021, Biz C et al. [20] performed a long-term follow-up comparison between the percutaneous technique described by Ma and Griffith and the Tenolig^®^ system, also performing an ultrasound-based check. The study highlighted the long-term validity of both techniques; however, with the Tenolig^®^ system, they observed a lower rate of inflammatory pathologies, a quicker return to sport and a greater degree of patient satisfaction. In 2008, a randomized prospective study conducted by Gigante A et al. [21] using SF-12 questionnaires found no statistically significant differences between open and percutaneous repair in the treatment of ATRs. Other authors have obtained similar results comparing the Tenolig^®^ system and open surgery [12]. Despite this, several meta-analyses have shown a better outcome in the case of percutaneous surgery [22,23]. However, more recent studies did not find statistically significant differences between the two groups [24].

In our study, there was a statistically significant difference between open and percutaneous surgery. In this study, Tenolig^®^ system repair led to better patient-reported clinical outcomes via ATRS at an average of 6 years of follow-up, despite a similar number of complications. To our knowledge, this is the first article which compares the clinical result 6 years later. The difference found in the functional score could be linked to the greater consideration of biology and the lower formation of adhesions.

ATRs can be career-ending injuries, since the Achilles tendon is crucial to the athlete, as it is essential for explosive activities such as running and jumping [25]. Overall, 61–100% of elite male athletes return to sports after ATRs [26]. However, few studies analyze the rate of return to sport in recreational athletes after ATRS. Lerch TD et al. found that 70% of patients were able to return to their previous sports activity level after the nonoperative treatment of ATRs [27]. At the 6-year follow up, about one in five patients no longer practiced sport after their injury. This percentage was similar between the two groups. However, almost one in two patients treated with the percutaneous technique returned to their pre-injury activity level compared to one third of patients treated with the open technique. According to the data of this study, all these characteristics make the percutaneous technique preferable to the open technique both in the sedentary population and in adults practicing non-professional sports.

Considering the number of total complications observed in the two groups, we observed a different percentage rate. In fact, we observed 12 total complications in the percutaneous group, equal to 11.65%, and 9 total complications in the open group, or 17.30%. The statistical evaluation of the total complications of the two samples presents a *p*-value of 0.46, and this result is therefore statistically not significant. The data agree with the current literature [28].

## 5. Conclusions

In conclusion, based on our results, the Tenolig^®^ repair system led to patient-reported clinical outcomes in long-term follow-up that were comparable to the population treated with the open technique. Complication rates were also comparable in the two samples examined.

The main problem of the percutaneous technique is currently the re-rupture rate. Although in our group under examination, a lower frequency of this event was observed compared to the literature, it remains essential to consider this eventuality above all from the perspective of the surgical indication and the type of patient to be treated. Patients with poor compliance or with a request to quickly return to sporting activity are not candidates for the percutaneous technique.

It is necessary to point out a notable difference in surgical time. The percutaneous technique, since it requires only two instances of minimally invasive access and has devices designed for the procedure, is significantly quicker. Furthermore, we must consider the fact that it is performed under local anesthesia, while the open technique requires spinal anesthesia.

We can therefore conclude, according to the data of this study, that the percutaneous technique is preferable in the sedentary and elderly populations, where a less invasive and quicker surgical procedure can be of greater benefit.

### Limitations of the Study and Future Directions

This study is limited by the systematic bias associated with retrospective studies and the numerical inhomogeneity of the samples compared. Furthermore, human error in obtaining the scores could have represented sources of bias. Further bias is given by the surgical indication provided by the first operator. Although these choices are motivated, most of the time, by the previously mentioned characteristics of the lesions, there is still a personal preference rate.

To further improve minimally invasive repair, the use of intraoperative ultrasonography assistance could be valid; in particular, we believe that this aid can significantly reduce errors in positioning the percutaneous device, consequently reducing the complication rate of this procedure.

## Figures and Tables

**Table 1:** This table summarizes the characteristics of the patients included.

Patients	Data
Number	155
Sex	143 males
	12 females
Age	48.30 ±14.75
Side	80 right 75 left
Type of Trauma	81 sports injuries 74 accidental events
Follow up	72.3 ± 31.6 3 months
Complications (no effect on function)	10 (6 superficial infections; 1 DVT *; 3 cases of temporary sural nerve palsy)
Complications (with effect on function)	11 (2 deep infections; 9 re-ruptures)

* DVT = deep vein thrombosis.

**Table 2:** Descriptive analysis of percutaneous and open groups.

Parameter	Percutaneous Surgery	Open Surgery	*p*-Value
Patients	103	52	–
(male, female)	(98, 5)	(45, 7)	
(left, right)	(51, 51)	(24, 28)	
Age (years)	48.9 ± 15.1	47.9 ± 14.5	
ASA **	1.5 ± 0.6	1.8 ± 0.5	
ATRS (points)	92.5 ± 10.0	82.0 ± 7.5	0.156
Procedure time (min)	19.4 ± 7.6	71.0 ± 26.7	<0.001
Follow-up duration (months)	73.1 ± 20.7	71.5 ± 18.5	
Complications	12 (11.6%)	9 (17.3%)	0.478
Without effect on function			
Superficial infections	2 (1.9%)	4 (7.7%)	0.183
Temporary sural nerve palsy	2 (1.9%)	1 (1.9%)	0.991
DVT *	1 (1.0%)	0 (0%)	
With effect on function			
Re-rupture	7 (6.8%)	2 (3.8%)	0.507
Deep infections	0 (0%)	2 (3.8%)	0.104

* DVT = deep vein thrombosis; ** ASA Physical Status Classification System.

## Data Availability

The data presented in this study are available on request from the corresponding author.
